# Unravelling the role of HAS2, GREM1, and PTGS2 gene expression in cumulus cells: implications for human oocyte development competency - a systematic review and integrated bioinformatic analysis

**DOI:** 10.3389/fendo.2024.1274376

**Published:** 2024-03-08

**Authors:** Ahmad Mohd Faizal, Marjanu Hikmah Elias, Norazilah Mat Jin, Muhammad Azrai Abu, Saiful Effendi Syafruddin, Ani Amelia Zainuddin, Nao Suzuki, Abdul Kadir Abdul Karim

**Affiliations:** ^1^ Department of Obstetrics & Gynecology, Faculty of Medicine, National University of Malaysia, Kuala Lumpur, Malaysia; ^2^ Faculty of Medicine & Health Sciences, Universiti Sains Islam Malaysia, Nilai, Negeri Sembilan, Malaysia; ^3^ Department of Obstetrics & Gynecology, Faculty of Medicine, Universiti Teknologi MARA, Jalan Hospital, Sungai Buloh, Selangor, Malaysia; ^4^ Medical Molecular Biology Institute, National University of Malaysia, Kuala Lumpur, Malaysia; ^5^ Department of Obstetrics & Gynecology, St Marianna School of Medicine, Kawasaki, Japan

**Keywords:** HAS2, PTGS2, grem1, cumulus cells, oocytes development competency, gene expression

## Abstract

The leading indicator for successful outcomes in *in-vitro* fertilization (IVF) is the quality of gametes in oocytes and sperm. Thus, advanced research aims to highlight the parameter in assessing these qualities – DNA fragmentation in sperm and oocyte development capacity (ODC) via evaluation of microenvironments involving its maturation process. Regarding oocytes, most evidence reveals the role of cumulus cells as non-invasive methods in assessing their development competency, mainly via gene expression evaluation. Our review aims to consolidate the evidence of GDF-9 derivatives, the HAS2, GREM1, and PTGS2 gene expression in cumulus cells used as ODC markers in relevant publications and tailored to current IVF outcomes. In addition to that, we also added the bioinformatic analysis in our review to strengthen the evidence aiming for a better understanding of the pathways and cluster of the genes of interest - HAS2, GREM1, and PTGS2 in cumulus cell level. Otherwise, the current non-invasive method can be used in exploring various causes of infertility that may affect these gene expressions at the cumulus cell level. Nevertheless, this method can also be used in assessing the ODC in various cohorts of women or as an improvement of markers following targeted tools or procedures by evaluating the advancement of these gene expressions following the targeted intervention.

## 1 Introduction

The success of *in-vitro* fertilisation (IVF) depends mainly on the quality of gametes, specifically the DNA quality of sperm and the overall quality of the oocytes (1). Most research focuses on enhancing oocyte quality to improve IVF outcomes. Clinically, various control ovarian stimulation protocols are used, targeting different gonadotrophin receptors, and timely oocyte collection is performed, with or without intracytoplasmic sperm injection, aiming for optimum fertilisation and high success rates per cycle (2). However, the developmental potential of oocytes following ovarian stimulation varies amongst women, leading to inconclusive overall IVF outcomes. In addition to research on oocyte quality, the culture media and IVF laboratory protocols evolved. Efforts have been exerted to improve media substances with targeted concentration to minimise stress in cultured embryos and promote better progression, leading to the production of good-quality embryos (3, 4). Despite these strategies, the current worldwide IVF success rate remains at approximately 35%–45% (5–7). To address this issue, researchers are exploring the development of oocyte quality. Various factors have been established to contribute to a better quality of the oocytes – mainly the level of hormones, such as growth hormone (GH) with insulin-like growth factor-1 (IGF-1). A recent study reported that higher follicular fluid levels of GH and IGF1 appear to be associated with better oocyte competency. Otherwise, the same study also reported no association of levels of TSH, fT3, fT4, 25OHD, or antithyroid antibodies in follicular fluid for oocyte quality (8). These hormonal levels can be altered due to dietary habits and the women’s body mass index (BMI). In obese women, ovarian inflammation leads to the production of pro-inflammatory cytokines and activation of cell death mechanism, resulting in ovarian tissue damage, microenvironment alteration via the releasing of oxidation stress, and alteration of microbiome metabolism. Not surprisingly, all these factors negatively impact both meiotic and cytoplasmic oocyte maturation – leading to poor oocyte quality (9). In addition to that, hyperinsulinemia also altered the endogenous pathway of steroidogenesis, which may have contributed to the imbalance of the intrinsic pathway in folliculogenesis, leading to suboptimal oocyte development, thus affecting its quality (10).

**Figure 1:**
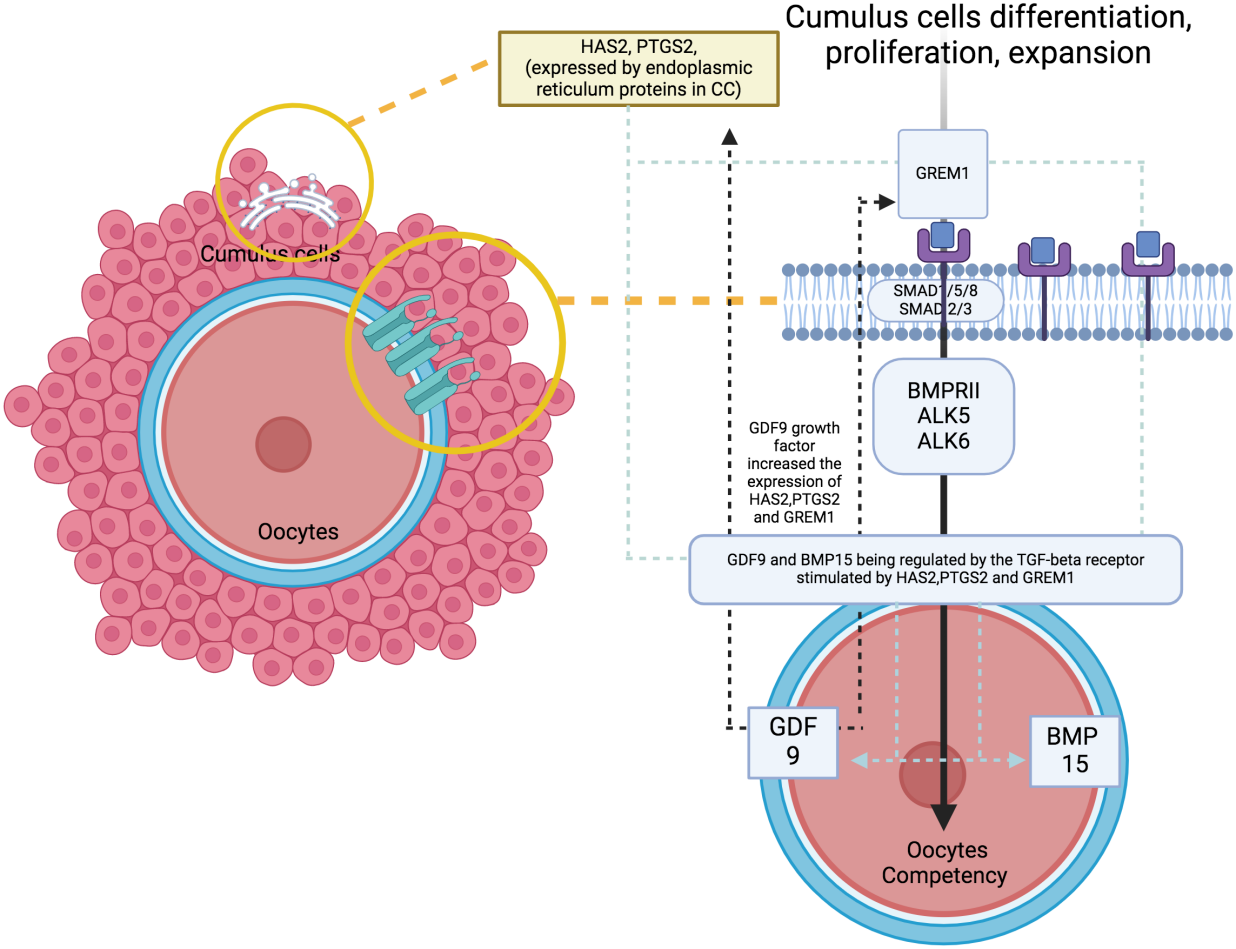
The signaling pathway with genes of interest expression and oocytes quality.

At the molecular level, the study of oocyte competency aims to predict embryo outcomes more effectively through the use of various biomarkers and signalling pathways (11). A promising non-invasive method involves analysing the cumulus cells (CC), because they can reflect the competency of developing oocytes. Apart from offering mechanical protection, CC plays a crucial role in providing paracrine signalling, metabolism pathways and overall gene regulation that promote oocyte development and maturation competency (12, 13). The CC differentiation, proliferation and expansion will lead to a better oocytes’ developmental regulation – the signaling pathway influence the oocytes quality (**Figure 1**). Therefore, promising evidence utilise cumulus-expressed genes as markers for assessing oocyte development competency, because they reflect the fundamental processes in the molecular environment (12–15). To date, several genes have been identified to coordinate the maturation of granulosa cells, leading to specific signalling pathways that contribute to CC expansion and promote oocyte development and maturation, primarily through growth differentiation factor 9 (GDF9) (16, 17). As established, GDF-9 is an oocyte-derived growth factor in the transforming growth factor β (TGF-β) superfamily. It plays a critical role in coordinating the expression of several genes that potentially contribute to oocyte and subsequent embryo quality (18, 19). These genes include Hyaluronan synthase 2 (HAS2), Prostaglandin-endoperoxide synthase 2 (PTGS2), Gremlin 1 (GREM1) and steroidogenic acute regulator protein (STAR) (20, 21). Distinctively, the available evidence strongly supports HAS2 and GREM1 as potential markers for CC in predicting oocyte competence compared with other genes. Additionally, oocyte nuclear maturation correlates with PTGS2, STAR, amphiregulin (AREG) and stearoyl-co-enzyme A desaturase 1 and 5 (SCD1 and SCD5), leading to a good CC expansion and development (22, 23). However, conflicting evidence reports that CC increases the expression of Pentraxin-related protein - PTX3, also known as TNF-inducible gene 14 protein (TSG-14), which is ultimately essential to correlate with oocyte maturation and influence its quality (24, 25). Regarding this matter, various genes and pathways are responsible for CC expansion, supporting oocyte development in animal and human models. However, the most established evidence suggests that specific GDF9 target genes, particularly HAS2, GREM1 and PTGS2, are closely correlated with the microenvironment in human oocytes (11, 12, 16, 23).

Understanding the molecular mechanisms underlying the microenvironment of oocyte development competency is crucial to address the variability in IVF success rates amongst women. Extensive efforts have been made to identify key genes and signaling pathways involved in CC expansion and maturation. Notably, HAS2, a critical enzyme responsible for hyaluronan synthesis, has been associated with increased cumulus expansion, indicating its potential as a biomarker for oocyte quality (26). Similarly, GREM1, an antagonist of bone morphogenetic proteins, plays a vital role in modulating the transforming growth factor-beta (TGF-β) pathway, thereby influencing CC function and oocyte developmental competence (12, 27). Additionally, PTGS2, an enzyme involved in prostaglandin synthesis, has been implicated in regulating ovulation, CC expansion and embryo implantation. Unravelling the interplay between these genes and their regulatory pathways provides valuable insights into the complex molecular environment surrounding oocyte development and may hold the key to accurately predict IVF success (28). In recent years, advancements in molecular techniques have provided researchers with the ability to explore gene expression patterns in CC with great precision and sensitivity (14, 23). Through analysing the gene expression profile of CC, researchers aim to identify novel biomarkers that can reliably predict oocyte developmental competence, potentially revolutionising current IVF practices. Additionally, studying the relationship between CC gene expression and oocyte developmental potential offers a non-invasive approach to assess oocyte quality before fertilisation. This approach spares patients from unnecessary invasive procedures and minimises the risk of embryo wastage. Moreover, gaining insights into the molecular basis of oocyte competence may pave the way for the development of personalised IVF treatment strategies, tailored to each individual’s specific molecular profile.

Therefore, in this review, we have compiled published data to elucidate the role of gene expressions of Hyaluronan synthase 2 (HAS2), Gremlin 1 (GREM1) and Prostaglandin-endoperoxide synthase 2 (PTGS2) in CC and their association with human oocyte development competency, particularly in terms of fertilisation and embryo development capacity (11, 13, 25, 27). Additionally, we will explore the involvement of these genes in relevant pathways using integrated bioinformatic analysis. By examining the constellation of these gene expressions and their correlated importance, we aim to consolidate the evidence supporting CC as an excellent biomarker that reflects oocyte competency. This approach will provide a better understanding of oocyte development competency at the molecular level.

## 2 Material and methods

### 2.1 Protocol registration

This systematic review adhered to the standard guideline protocol based on the Preferred Reporting Items for Systematic Reviews and Meta-Analysis (PRISMA) (29). It was registered in the international database of prospectively registered systematic reviews known as PROSPERO with the registration number CRD42023413686 (30).

### 2.2 Information source and search strategy

The publications within 20 years (2003-2023) were searched using keywords in PubMed and ScienceDirect [All Fields]: ‘*In vitro* fertilization’ OR IVF OR ‘*in vitro* maturation’ OR IVM AND ‘gene expression’ AND HAS2 OR ‘hyaluronan synthase 2’ OR PTGS2 OR ‘prostaglandin-endoperoxide synthase 2’ OR COX2 OR GREM1 OR ‘gremlin 1’ AND ‘cumulus cell’ AND ‘oocytes development competency’ OR fertilization OR blastulation OR biochemical OR ‘clinical pregnancy’ OR ‘live birth’ OR ‘oocyte quality’ OR ‘embryo quality.’ Whereas in SCOPUS, the keywords used – [All Fields]: ‘*in vitro* fertilization’; OR ivf OR ‘*in vitro* maturation’ OR ‘ivm’ AND [All Fields]: gene AND expression AND [All Fields]: has2 OR ‘hyaluronan synthase 2’; OR ptgs2 OR ‘prostaglandin-endoperoxide synthase 2’; OR cox2 OR grem1 OR ‘gremlin 1’ AND [All Fields]: ‘cumulus cell’ AND [All Fields]: ‘oocytes development competency’ OR fertilization OR blastulation OR biochemical OR & ‘clinical pregnancy’; OR AND ‘live birth’ OR ‘oocyte quality’; OR ‘embryo quality’ AND [All Fields]: ‘infertile women’. Subsequently, all the included studies references were screened for duplication using EndNote^®^ version 20.0.1. The search was improved by manual search using the reference lists from selected articles.

### 2.3 Study selection, data extraction and risk of bias assessment

Based on an initial search, fives authors (A.M.F, A.M.A, M.H.I, M.J.N and A.K.A.K.) screened all titles and abstracts of potential manuscripts. The selection criteria included manuscripts published in English from January 2012 to December 2022, evaluating the expression of three specific genes of interest - Hyaluronan synthase 2 (HAS2), Gremlin 1 (GREM1) and Prostaglandin-endoperoxide synthase 2 (PTGS2) - in CC of human oocytes. Following the title and abstract screening, full-text screening was conducted, excluding manuscripts that used different genes, non-CC as experimental material, non-English language, case reports and review articles involving non-human subjects. The remaining potential manuscripts were then independently reviewed. The final selected manuscripts provided a detailed study design, focusing on the expression of all three genes of interest - Hyaluronan synthase 2 (HAS2), Gremlin 1 (GREM1) and Prostaglandin-endoperoxide synthase 2 (PTGS2) - in human CC and correlated these gene expressions with oocyte development competency as the primary outcome. The conflicts in selection amongst authors were resolved through detailed discussions and opinions provided by the fifth, sixth and seventh authors (S.S.E, N.S. and A.A.Z). Additionally, the National Institutes of Health (N.I.H.) tool for observational studies was employed to assess the quality of the selected manuscripts. This evaluation was based on 14 variables, with a scoring system of 1 for ‘yes,’ 0 for ‘no,’ or ‘non-applicable’ for N.A. The manuscripts were then categorised as poor (0–5), fair (6–9) or good (10–14) based on their total scores (**Table 1**). Overall, the included studies in our review achieved a minimum fair to good score. Subsequently, the final data were extracted and organised based on the authors’ last names, year of publication, country, study design, type of cohort and number of samples (if applicable). Additionally, each study’s gene expression and its correlation with oocyte development competency were tabulated as the main outcome (**Table 1**).

**Table 1:** Summary of Included Studies.

No	Author/Year	Country	Study Design	No sample	Gene of Interest	Outcome Measures	Oocytes Maturation Rates	Fertilization Rates	Embryo Development Rate	Degree of fragmentation	Proportion of good quality embryos
1	Adriaenssens 2010 (31)	Belgium	Prospective IVF Cohort	63	ALCAM PTGS1 PTGS2 HAS2 VCAN SDC4 GREM1 SPROUTY4 RPS6KA2 DUSP16	The study assessed the patient characteristics – age BMI, ovarian stimulation dependent variables rFSH and HMG - HP and oocyte developmental competence related variables with subsequently correlated it with genes of interest as the main outcome.	VCAN expression levels were significantly correlated with oocytes maturity (p<0.005) and not significantly with DUSP16 (n.s) No correlation with HAS2, PTGS2 and GREM1	HAS2 expression levels were correlated with 2PN (p<0.1) No correlation with PTGS2 and GREM1	ALCAM and PTGS2, GREM1 expression levels were significantly correlated with >7 cells D3 (p<0.05) and not significantly with SPROUTY4 No correlation with HAS2	RPS6KA2 expression levels were significantly correlated with low fragmentation and not significantly with SDC4 No correlation with HAS2, PTGS2 and GREM1	No correlation with HAS2, PTGS2 and GREM1
2	L.J McKenzie 2004 (32)	USA	Prospective IVF Cohort	108	GREM1 HAS2 PTGS2 RRNA18S	The study assessed the human cumulus granulosa cell gene expression in a predicting the fertilization and embryo selection in women undergoing IVF	HAS2 expression is predictive of oocyte maturity (p<0.05) Increase 5.2-fold relative expression GREM1 - sensitivity and specificity for oocyte maturity 63 and 93%	PTGS2 and GREM1 are predictors of fertilization (p<0.05) Increase 5.2-fold relative expression GREM1 - sensitivity and specificity for fertilization 72 and 78%,	PTGS2 and GREM1 are predictive of embryo development (p<0.05) Increase 5.2-fold relative expression GREM1 - sensitivity and specificity for embryo quality 83 and 81%	N/A	PTGS2 expression is 6-fold higher, GREM1 expression is 15- fold higher and HAS2 expression is 6-fold higher in good grade embryos
3	Nona Mishieva 2020 (33)	Russia	Prospective IVF Cohort Assessing oocytes competency infertility women using DuoStim approached in decrease ovarian reserved (DOR) women	39	GREM1 HAS2 PTGS2 VCAN ALCAM IPTKA TRPM7 SDC4 CALM2 SPSB2 TP53I3 PGR PFKP	The study assessed the oocytes competency among infertility women using DuoStim approached in decrease ovarian reserved (DOR) women	The expression levels of VCAN, SDC4 and TP53I3 significantly higher group 2 LPS patients VCAN (15.542 ± 6.8 versus 20.353 ± 10.58; P = 0.001), SDC4 (1.016 ± 0.65 versus 1.318 ± 0.97; P = 0.013), TP53I3 (0.185 ± 0.09 versus 0.270 ± 0.11; P <0.05) VCAN not correlate with oocyte maturation 10 out of 13 had similar levels of expression including HAS2, PTGS2 and GREM1 in both group (P>0.05)	Comparable expression of HAS2, PTGS2 and GREM1 in both group No correlation with HAS2, PTGS2 and GREM1 (P>0.05)	Comparable expression of HAS2, PTGS2 and GREM1 in both group No correlation with HAS2, PTGS2 and GREM1 (P>0.05)	N/A	VCAN not correlate with embryo quality Comparable expression of HAS2, PTGS2 and GREM1 in both group No correlation with HAS2, PTGS2 and GREM1 (P>0.05)
4	Giovanni Coticchio 2017 (34)	Israel	Prospective IVM Cohort	Not state	3BHSD ADAMTS AREG BMPR2 CYP11A1 CYP19A1 ERBB2 EREG FSHR GJA1 GPX3 GREM1 HAS2 LHR NOS2 PTGS2 SFRP4 SFRP5 STAR UGP2	The study assessed the differential regulation of cumulus cell transcription during oocyte maturation *in vivo* and *in vitro*	LHR gene overexpressed in both IVM-GV and IVM-MII COCs. The AREG was increased in IVM-MII COCs, low in COCs matured *in vitro* (IVM-GV) EREG expression was 4-fold higher in IVM-MII COCs. GREM1 expression had 4-fold increase in COCs matured *in vitro*. The BMPR2 mRNA levels were moderately increased in IVM-GV COCs. Post IVM, the SFRP4 was 8-fold > IVF-MII and IVF-MII COCs and NOS2 increased in IVM COCs SFRP5 mRNA were 2-fold lower in COCs matured *in vitro* and CYP11A1, 3-fold more COCs matured *in vitro* HAS2 gene reduced in COCs collected from hCG-primed IVM cycles - IVM-MII group. The PTGS2 reduced in both IVM-GV and IVM-MII COCs. GPX3 and UGP2 were dysregulated following IVM. GJA1 was 14- and 8-fold more expressed in IVM-GV and IVM-MII COCs	N/A	N/A	N/A	N/A

N/A is Non Applicable.

### 2.4 Integrated bioinformatic analysis

**Figure 2:**
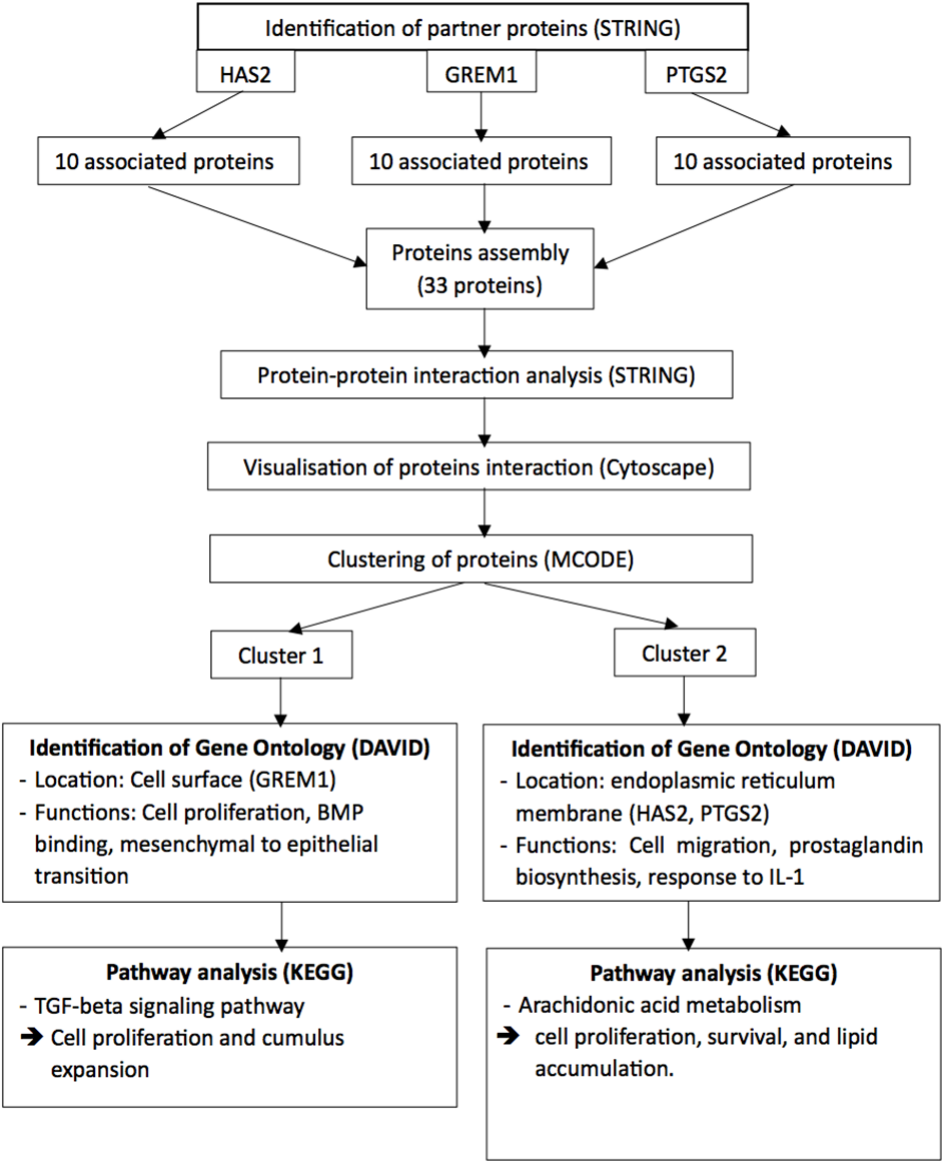
The Integrated Bioinformatic Analysis and Work Flow.

To identify the possible pathways related to HAS2, GREM1 and PTGS2, all the proteins interaction with these gene product were identified. The predicted functional partners for HAS2, GREM1 and PTGS2 were identified separately using the STRING software (https://string-db.org/). Subsequently, all the proteins interacting with HAS2, GREM1, and PTGS2 were gathered, and their protein-protein interactions were once again identified using the STRING software. The results from STRING were then exported to the Cytoscape software (http://www.cytoscape.org/) to visualise the molecular interaction networks and integrate gene-expression profiles of the included genes. To analyse the target network and identify protein clusters, the MCODE plug-in was utilised with the following settings: degree cut-off = 2, node density cut-off = 0.1, node score cut-off = 0.2, K-score = 2, and max depth = 100). All the clusters were analysed using the Database for Annotation, Visualisation and Integrated Discovery (DAVID) to explore the gene ontology with significant functional-annotation enrichment related to oocyte development (35). Subsequently, the Kyoto Encyclopaedia of Genes and Genomes (KEGG) pathway was employed to reveal the involvement of genes in pathways associated with oocyte development (36) (**Figure 2**).

## 3 Results

### 3.1 Search sequence and quality assessment

**Figure 3:**
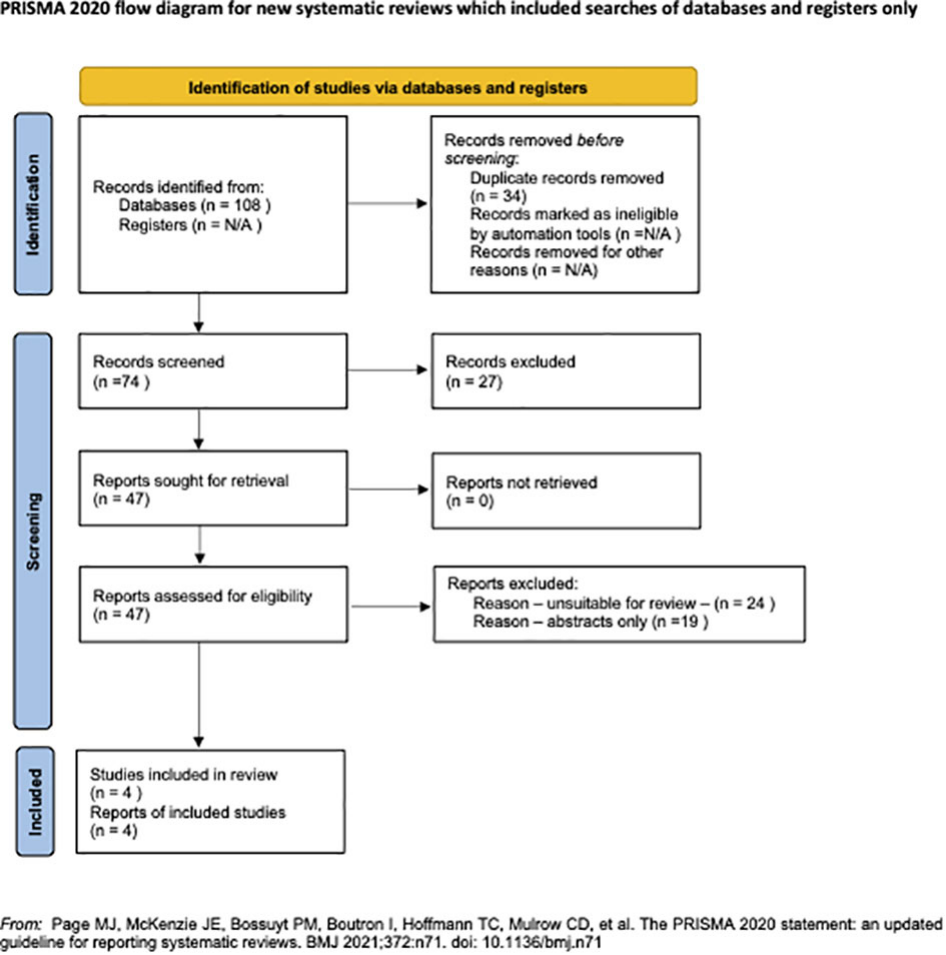
The PRISMA Flow Chart.

A total of 108 studies were retrieved during the primary search (**Figure 3**). After removing 34 duplicates, the remaining 74 articles were thoroughly screened based on our inclusion criteria. Amongst them, 27 articles were excluded, leaving 47 for full-text evaluation. After a detailed evaluation, 43 articles were further discarded: 24 were deemed unsuitable, and 19 were poster presentations or conference abstracts without detailed results. Subsequently, four studies that focused on oocyte development capacity parameters were selected for this review. To ensure quality and minimize bias, all selected articles were evaluated using the National Institutes of Health (N.I.H.) tool for observational studies. Notably, all four articles obtained a good score, indicating a low risk of bias (**Supplementary Table 1**).

### 3.2 Studies characteristics

This review included a total of 210 infertility women who underwent IVF, with studies using at least three genes of interest - HAS2, GREM1 and PTGS2 - to correlate with oocyte development competency (ODC) outcomes. The ODC parameters, mainly oocyte maturation rate, fertilisation rate and embryo developmental rate (at least at the 7-cell stage on Day 3 or blastocyst formation following sperm injection), were recorded in all the included studies. The quality of the embryos was assessed based on the degree of fragmentation rates, if applicable, and the excellent quality of embryos was also considered the main outcome. Amongst the included studies, two focused on standard infertility women cohorts undergoing IVF, whilst one focused on decreased ovarian reserve women (31–33). Additionally, one study focused on ODC in an *in-vitro* maturation (IVM) cohort, comparing the expression of genes of interest in *in-vivo* and *in-vitro* maturation settings (34). All the relevant information from the included studies has been summarized in **Table 1**.

### 3.3 Main outcomes

#### 3.3.1 Oocytes maturation rates

One study agreed that GREM1 higher expression was correlated with better oocyte maturation compared to the other two genes, with sensitivity and specificity for oocyte maturity at 63% and 93%, respectively (32). The same study found that HAS2 also significantly predicts oocyte maturation (32). However, in the IVM cohort, the HAS2 expression was reduced in CC in hCG-primed IVF cycles (34). Otherwise, PTGS2 expressions were comparable in all the studies for oocyte maturation rate (OMR), except for the IVM cohort, which showed a reduction in IVM-GV and IVM-MII CC (31, 34). The expression of HAS2, PTGS2, and GREM1 was low and similar in both follicular-phase-derived and luteal-phase-derived oocytes treated with double stimulation, and there was no association with the outcome in all parameters of interest - OMR, FR, EDR, and GQE (33).

#### 3.3.2 Fertilisation rates

Similarly, for FR, only one study concurred that GREM1 and PTGS2 expression was correlated with better FR compared with the two other genes, with sensitivity and specificity for fertilisation at 72% and 78%, respectively (32). However, one study found a significant correlation between HAS2 expression and 2PN (two pronuclei) fertilisation outcome (31). Meanwhile, a similar expression of HAS2, PTGS2 and GREM1 was found in both follicular-phase-derived and luteal-phase-derived oocytes treated with double stimulation for FR (33).

#### 3.3.3 Embryo development rates

Regarding EDR, one study revealed that an increase in 5.2-fold relative expression of GREM1 predicted good embryo quality development, with sensitivity and specificity of 83% and 81%, respectively. Additionally, higher PTGS2 expression also significantly predicts good embryo development, similar to GREM1 (32). However, all studies revealed low expression levels of HAS2 that does not correlate with EDR. Conversely, a similar expression of HAS2, PTGS2 and GREM1 was found in both groups follicular-phase-derived and luteal-phase-derived oocytes treated with double stimulation for EDR (33). As for the degree of fragmentation, only one study included it in their outcome, where they found that the degree of fragmentation does not correlate with HAS2, PTGS2 and GREM1 expression in CC (31).

#### 3.3.4 Proportion of good quality of embryo

The genes of interest were tabulated based on their fold expression correlating with the proportion of good-grade embryos (GQE). GREM1 exhibited the highest expression fold, being 15-fold higher, whilst PTGS2 and HAS2 showed at least six-fold higher expression in good-grade embryos (32). Furthermore, similar expression of HAS2, PTGS2 and GREM1 was found in both groups follicular-phase-derived and luteal-phase-derived oocytes treated with double stimulation for GQE (33). However, one study found no correlation between all genes of interest - HAS2, PTGS2 and GREM1 - with GQE (31).

### 3.4 Pathways analysis via protein–protein interaction network and modular analysis.

**Figure 4:**
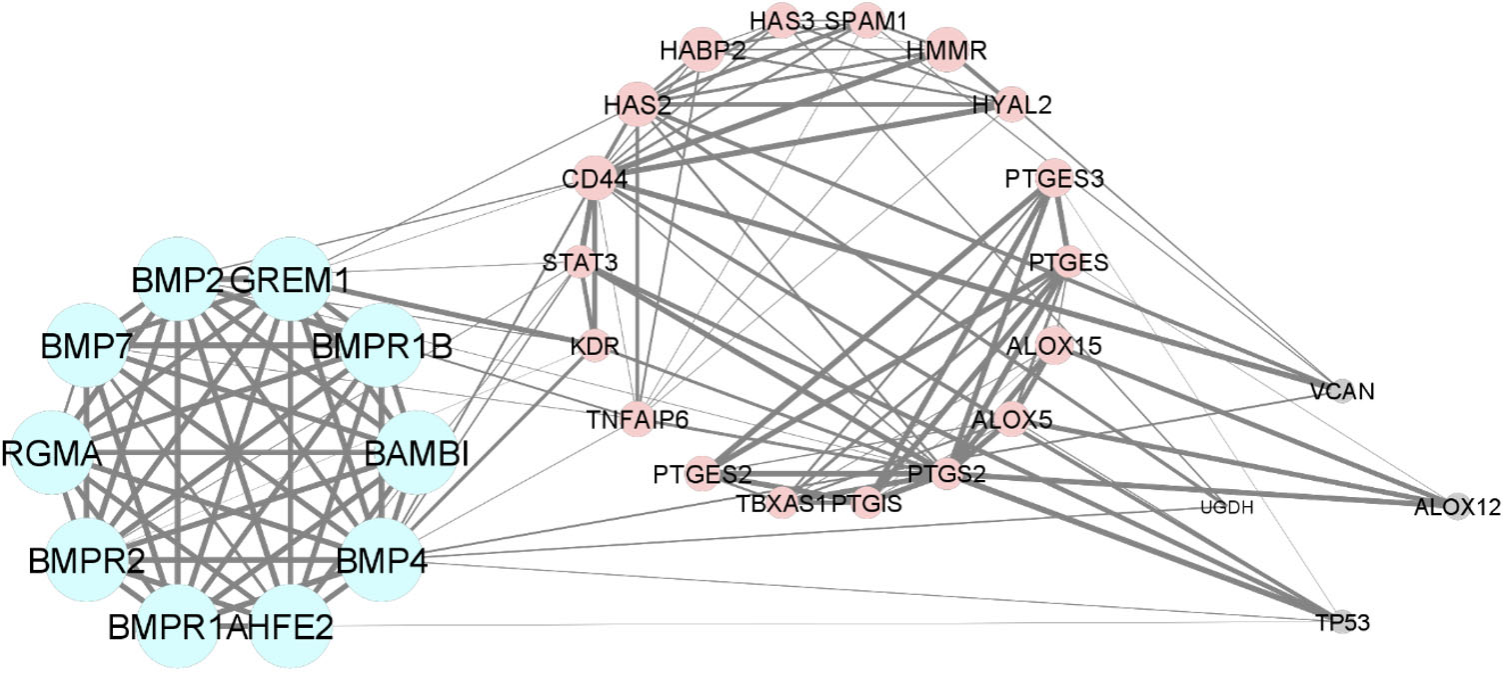
The Protein-protein interaction and clustering of HAS2, GREM1, PTGS2 and their functional partners.

In the bioinformatic analysis, a total of 30 predicted functional partner proteins associated with HAS2, GREM1 and PTGS2 were identified. These genes were then subjected to a protein-protein interaction (PPI) network analysis, resulting in a complex network containing 32 nodes and 139 edges. The PPI network showed a highly significant enrichment with a p-value of <1.0e^−16^ and an average local clustering coefficient of 0.707. To visualise the molecular interactions, the network data from STRING was transferred to Cytoscape software. Additionally, using the molecular complex detection algorithm (MCODE), two significant modules were identified within the PPI network complex (**Figure 2**). Functional-annotation clustering revealed that cluster 1 comprised 10 nodes and 25 edges (score = 10), whilst cluster 2 consisted of 18 nodes and 59 edges (score = 6.941). The clustering of all the genes in is based on the gene’s cellular location, biological functions, pathways and molecular functions (**Figure 4**).

### 3.5 Gene ontology and pathway enrichment of the clusters

The GO and pathway enrichment analyses revealed that GREM1 is clustered in cluster 1. GREM1 and its functional partner proteins are localised at the cell surface (GO:0009986) and in the extracellular space (GO:0005615). These proteins were found to be involved in the TGF-beta signalling pathway (hsa04350), which includes the bone morphogenic protein (BMP) signalling pathway (GO:0030509), Hippo signalling pathway (hsa04390) and cytokine–cytokine receptor interaction (hsa04060) through BMP binding (GO:0036122). Additionally, the proteins in cluster 1 are also involved in positive regulation of transcription from RNA polymerase II promoter (GO:0045944). HAS2 and PTGS2 are clustered in cluster 2. These proteins and their functional partners are mostly localised at the endoplasmic reticulum membrane (GO:0005789). PTGS2 is involved in arachidonic acid metabolism (hsa00590) and participates in prostaglandin biosynthetic process (GO:0001516). Meanwhile, HAS2 is involved in positive regulation of cell migration (GO:0030335) and response to inflammation through the cyclooxygenase pathway (GO:0019371) and cellular response to interleukin-1 (GO:0071347). **Table 2** displays the Functional annotation clustering for clusters 1 and 2, focusing on GREM1, HAS2 and PTGS2 (**Table 2**).

**Table 2:** Functional annotation clustering of HAS2, GREM1, PTGS2 and their functional partners.

Cluster	Term	Description	Genes	*p*-value
1	CC_GO:0005615	extracellular space	*GREM1, BMP4, BMP2, BMPR2, HFE2, BMP7*	6.83 X10^-4^
	CC_GO:0009986	cell surface	*GREM1, BMP2, BMPR2, HFE2, RGMA*	1.01 X10^-4^
	hsa04350	TGF-beta signaling pathway	*GREM1, BMP4, BMP2, BMPR2, BAMBI, HFE2, BMPR1B, BMP7, BMPR1A, RGMA*	2.54 X10^-8^
	BP_ GO:0045944	positive regulation of transcription from RNA polymerase II promoter	*GREM1, BMP4, BMP2, BMPR2, HFE2, BMPR1B, BMP7, BMPR1A, RGMA*	1.86 X10^-9^
	BP_ GO:0008284	positive regulation of cell proliferation	*GREM1, BMP4, BMP2, BAMBI*	1.68 X10^-3^
	MF_ GO:0036122	BMP binding	*GREM1, BMPR2, HFE2, BMPR1B, BMPR1A*	1.14X10^-10^
2	CC_ GO:0005789	endoplasmic reticulum membrane	*PTGIS, TBXAS1, HAS2, PTGS2, PTGES*	0.011
	hsa01100	Metabolic pathways	*PTGIS, PTGES2, ALOX5, HYAL2, PTGES3, TBXAS1, ALOX15, PTGS2, SPAM1, PTGES*	9.79X10^-5^
	hsa00590	Arachidonic acid metabolism	*PTGIS, PTGES2, ALOX5, PTGES3, TBXAS1, ALOX15, PTGS2, PTGES*	1.46X10^-12^
	BP_ GO:0001516	prostaglandin biosynthetic process	*PTGIS, PTGES2, PTGES3, TBXAS1, PTGS2, PTGES*	3.16X10^-12^
	BP_GO:0030335	positive regulation of cell migration	*TNFAIP6, STAT3, KDR, HAS2*	1.45X10^-3^
	BP_ GO:0019371	cyclooxygenase pathway	*PTGIS, PTGES2, PTGES3, TBXAS1, PTGS2*	5.11X10^-11^
	BP_ GO:0071347	cellular response to interleukin-1	*PTGIS, HYAL2, HAS2*	2.42X10^-3^

## 4 Discussion

As established, the critical development of oocyte maturation occurs in the cumulus oocyte complex (37) during the follicular phase (38). Optimal expansion of CC is crucial to ensure better oocyte quality. Therefore, the oocyte developmental competency (ODC) can be assessed at the CC level based on gene expression (12, 13). Numerous studies have utilised GDF-9 derivatives such as Prostaglandin-endoperoxide synthase 2 (PTGS2), gremlin1 (GREM1), hyaluronic acid synthase 2 (HAS2) and pentraxin 3 (PTX3) as markers for ODC, as they reflect the immediate ODC during CC expansion (11, 18, 19). In this review, we found that GREM1 expression is an important marker for oocyte maturation rates (OMR) compared to both HAS2 and PTGS2 (32). Healthy oocytes tend to increase the expression of GREM1 in their CC, promoting folliculogenesis and enhancing spindle activities, ultimately leading to oocyte maturation (39, 40). The majority of the evidence aligns with these findings, consolidating the importance of GREM1 in OMR (12, 13, 17). Our review found that following rescue IVM, PTGS2 expression is reduced, indicating that proper IVM should not be preceded by hCG injection because it may interfere with oocyte maturation rates (34). Therefore, recent evidence recommends pre-IVM culture, as well as standard IVM procedures without hCG, to improve the microenvironment at the CC expansion level, aiming for a better OMR following IVM culture (41). Furthermore, our review revealed that GREM1 is also considered a predictor of good fertilisation rates (FR) and EDR (32). With optimum GREM1 expression following maturation, meiosis II (MII) oocytes can fertilise following insemination by sperm and subsequently undergo embryo development (42). The gathered evidence suggests that a reduction in GREM1 does affect the fertilisation rates and EDR, particularly in suboptimal cohorts, such as decrease ovarian reserved (DOR), or in cases of poor oocyte quality in women with endometriosis or polycystic ovarian syndrome (13, 43, 44).

Besides that, the expression of HAS2 in CC was associated with higher 2PN fertilisation (31). Most evidence postulates that an increase in the fold of HAS2 expression significantly correlates with better oocyte quality, leading to an increase in fertilisation (11, 16, 27, 32). Moreover, the unique expression of HAS2 is synergistically influenced by hCG or LH as maturation triggers (26). Therefore, standard IVM cycles without hCG will incorporate LH in the culture media to enhance HAS2 expression, aiming for better oocyte quality, fertilisation rates (FR) and EDR. Furthermore, our review agrees that the proportion of good quality of embryo (GQE) is influenced by all GDF-9 derivative gene expressions (32, 33). However, a higher fold of GREM1 expression is associated with an increase in the GQE cohort (32). In exploring the GDF-9 derivatives, most publications strongly support the importance of these three genes - HAS2, PTGS2 and GREM1 - in modulating embryo development (45, 46). Numerous pieces of evidence demonstrate that higher median expression of GDF-9 downstream predicts better quality embryo outcomes (11, 15, 19, 27, 40). The regulation of human follicles through GDF-9 has been well-established for decades. Any deviation or mutation in its molecular regulation can lead to possible subfertility, mainly due to a blockage of the early follicular phase, resulting in disruption of the meiosis process. Additionally, subsequent evidence suggests that GDF-9 also influences the later stages of oocyte development (47). Therefore, by dissecting the mechanisms of their regulation, a better understanding of the maturation of ongoing dominant follicles can be achieved. CC expansion is initiated by the secretion of GDF-9, with HAS2 playing a crucial role in promoting expansion through the production of hyaluronic acid. Subsequently, PTGS2 further promotes expansion by secreting prostaglandin E2. Both mechanisms are considered crucial for maturation and ovulation. Defects in these mechanisms, as observed in animal models, result in defects in ovulation, fertilisation, embryo quality and implantation (48). As for GREM1, it acts by inhibiting the signalling for BMP rather than GDF-9. During cumulus expansion, GREM1 promotes mural granulosa cell luteinisation, mostly during the oocyte maturation phase (22). Therefore, all three of these genes influence CC expansion, thus reflecting the ODC in the human reproductive field (49).

From the bioinformatic analysis, GREM1 is clustered in cluster 1, whilst HAS2 and PTGS2 are separated in cluster 2. GREM1, along with its functional partner proteins, such as BMPR2, BMP2 and HFE2, is located in the extracellular space and cell surface, indicating its involvement in interactions with the cellular environment and adjacent cells. Communication is crucial for oocyte development. The cumulus-oocyte complex’s development is primarily driven by short-distance communication through extensive local cell-to-cell interactions, often involving members of the transforming growth factor-beta (TGF-β) family, such as inhibin, activin, anti-Müllerian hormone and growth and differentiation factor 9 (GDF9) (50). GREM1, being a downstream target protein of GDF9, plays a pivotal role in regulating the crosstalk between GDF9 signalling and BMP signalling pathways. It selectively inhibits the differential effect of the BMP signal whilst preserving the GDF9 signal. This unique regulation may promote luteinisation of ovarian granulosa cells whilst supporting CC expansion in the ovarian follicle (51). The proteins in cluster 1 also have roles in positive regulation of transcription from RNA polymerase II promoter and positive regulation of cell proliferation. GREM1, BMP4, BMP2 and BAMBI are associated with promoting cell proliferation, suggesting their potential roles in follicular development and CC expansion.

In contrast to GREM1, the proteins HAS2 and PTGS2 in cluster 2 are localised within the endoplasmic reticulum membrane. This cellular localisation suggests their involvement in cellular compartmentalisation and localisation processes within the oocyte. Both HAS2 and PTGS2 are integral components of key metabolic pathways. In particular, they play crucial roles in the arachidonic acid metabolism pathway, which is important for the production of bioactive lipid mediators, including prostaglandins. The prostaglandin biosynthetic process, enriched by HAS2 and PTGS2, is of particular significance because prostaglandins influence *in vitro* maturation of oocytes (52). PTGS2’s involvement in the cyclooxygenase pathway further emphasises its significance in prostaglandin synthesis, contributing to various reproductive processes. Moreover, the gene ontology terms associated with HAS2, such as positive regulation of cell migration, indicate its potential involvement in CC expansion, a critical process for successful oocyte maturation. Moreover, the enrichment of cellular response to interleukin-1 by HAS2 suggests its possible role in responding to inflammatory signals during oocyte development. Notably, exposure to high levels of IL-10 and IL-1β has been reported to decrease CC expression of GREM1, indicating that inflammation in the follicular fluid, particularly amongst obese women, could impact the cumulus oocyte complex and alter its microenvironment, significantly influencing GREM1 gene expression (44). Overall, the CC have demonstrated that GDF9 upregulates the expression of essential genes, including HAS2, PTGS2, GREM1 and STAR, whilst concurrently downregulating luteinising hormone receptor, a crucial factor for follicle development and cumulus expansion. The regulation of GDF9 and its downstream factors in CC presents a promising potential as an indicator of oocyte quality (12). Understanding these intricate molecular interactions offers valuable insights into oocyte development and maturation, which could lead to significant advancements in reproductive medicine and fertility treatments.

## 5 Conclusion

Our review summarizes the gene expression results of GDF-9 derivatives in CC, which can be reliable predictors in IVF, primarily in OMR, FR, EDR, and GQE. However, CC predictors are constellations of evidence rather than individuals, as different gene expressions represent different IVF parameters based on our review. Our analysis of the literature and bioinformatics provides evidence of a potential link between the expression levels of GREM1, PTGS2, and HAS2 in cumulus cells and the maturation of the oocyte and subsequent fertilization rates and embryo development. However, the link has yet to be consistently confirmed across all studies. Further research is necessary to verify the relevance of this connection. Our study has certain limitations, as the included papers did not correlate the outcomes with live birth rates. Hence, the molecular conclusions are limited to gene expression alone. Nonetheless, these candidate genes’ expression levels can be a valuable indicator correlated with IVF outcomes, predicting good embryo development and quality. These findings can help improve overall IVF outcomes.

## Author contributions

AF: Conceptualization, Data curation, Formal analysis, Investigation, Methodology, Writing – original draft. ME: Conceptualization, Data curation, Formal analysis, Methodology, Software, Writing – original draft. NJ: Conceptualization, Formal analysis, Methodology, Resources, Writing – review & editing. MA: Conceptualization, Data curation, Formal analysis, Investigation, Methodology, Writing – review & editing. SS: Conceptualization, Data curation, Formal analysis, Methodology, Supervision, Writing – review & editing. AZ: Data curation, Formal analysis, Supervision, Writing – review & editing. NS: Data curation, Formal analysis, Methodology, Supervision, Validation, Writing – review & editing. AK: Project administration, Supervision, Writing – review & editing.
