# Supplementary material for: Unravelling the role of HAS2, GREM1, and PTGS2 gene expression in cumulus cells: implications for human oocyte development competency - a systematic review and integrated bioinformatic analysis
Source: Front Endocrinol (Lausanne). 2024 Mar 8;15:1274376. doi: 10.3389/fendo.2024.1274376 (PMC10957552; doi:10.3389/fendo.2024.1274376)
Supplement: Supplementary file 1 [file Table_1.pdf]

Supplementary Table I : NIH Quality Assessment Tool

No	NIH Quality Assessment Tool	Adriaenssens et al 2010	L.J McKenzie et al 2004	Nona Mishieva et al 2020	Giovanni Coticchio et al 2017
1	Was the research question or objective in this paper clearly stated?	Y	Y	Y	Y
2	Was the study population clearly specified and defined?	Y	Y	Y	Y
3	Was the participation rate of eligible persons at least 50%?	Y	Y	Y	Y
4	Were all the subjects selected or recruited from the same or similar populations (including the same time period)? Were inclusion and exclusion criteria for being in the study prespecified and applied uniformly to all participants?	Y	Y	N	Y
5	Was a sample size justification, power description, or variance and effect estimates provided?	N/A	N/A	N/A	N/A
6	For the analyses in this paper, were the exposure(s) of interest measured prior to the outcome(s) being measured?	Y	Y	Y	Y
7	Was the timeframe sufficient so that one could reasonably expect to see an association between exposure and outcome if it existed?	Y	Y	Y	Y
8	For exposures that can vary in amount or level, did the study examine different levels of the exposure as related to the outcome (e.g., categories of exposure, or exposure measured as continuous variable)?	Y	Y	Y	Y
9	Were the exposure measures (independent variables) clearly defined, valid, reliable, and implemented consistently across all study participants?	Y	Y	Y	Y
10	Was the exposure(s) assessed more than once over time?	N/A	N/A	N/A	N/A
11	Were the outcome measures (dependent variables) clearly defined, valid, reliable, and implemented consistently across all study participants?	Y	Y	Y	Y
12	Were the outcome assessors blinded to the exposure status of participants?	N/A	N/A	N/A	N/A
13	Was loss to follow-up after baseline 20% or less?	Y	Y	Y	Y
14	Were key potential confounding variables measured and adjusted statistically for their impact on the relationship between exposure(s) and outcome(s)?	Y	Y	Y	Y
**TOTAL (/14)** (Y = Yes, N=No, NA=Non applicable)		11	11	10	11
**Quality Assessment Score** Poor (0-5), fair (6-9), or good (10-14)		Good	Good	Good	Good
